# Castleman's Disease: An Intrapulmonary Form with Intrafissural Development

**DOI:** 10.1100/tsw.2009.110

**Published:** 2009-09-14

**Authors:** Hajer Racil, Sana Cheikh Rouhou, Olfa Ismail, Saoussen Hantous-Zannad, Nawel Chaouch, Mourad Zarrouk, Belhassen Smati, Faouzi Mezni, Abdellatif Chabbou

**Affiliations:** ^1^ Pulmonology Department, Pavillon II, Abderrahman Mami Hospital, Ariana, Tunisia; ^2^ Pathology Department, Abderrahman Mami Hospital, Ariana, Tunisia; ^3^ Radiology Department, Abderrahman Mami Hospital, Ariana, Tunisia; ^4^ Thoracic Surgery Department, Abderrahman Mami Hospital, Ariana, Tunisia

**Keywords:** Castleman, angiofollicular hyperplasia, pulmonary, interlobar fissure

## Abstract

Castleman's disease (CD) is an uncommon, mainly benign, lymphoproliferative disorder of unknown etiology, mostly involving the mediastinum. Parenchymal lung involvement of the disease is exceedingly rare. We describe a case of CD in a 23-year-old woman with a 4-year history of recurring dyspnea and nonproductive cough, whose chest X-ray showed an abnormal shadow of the right hilum. Chest computed tomography confirmed the presence of a tissue-density mass of the right lower lobe, demonstrating poor contrast enhancement, associated with multiple laterotracheal and mediastinal lymphadenopathies. The patient underwent curative surgery, revealing a right hilar compressive mass, with an intrafissural development between the superior and middle lobes. Pneumonectomy was performed due to profuse bleeding. This case of CD is particular because of its unusual intrapulmonary location and its intrafissural development. Poor contrast enhancement is atypical in CD.

## BACKGROUND

Castleman's disease (CD), also known as angiofollicular lymph node hyperplasia, was first described in 1954 by Dr. Benjamin Castleman at Massachusetts General Hospital[1].

CD is an uncommon, mainly benign, lymphoproliferative disorder of unknown etiology, mostly involving the mediastinum[2]. CD could be categorized into two clinical types: unicentric (or localized form) and multicentric (or systemic form)[3,4]. Three distinct histological variants are recognized: the hyaline vascular (HV) type (seen in 90% of cases), the plasma cell (PC) type (8–9% of cases), and an intermediary "mixed" type (1–2% of cases)[3,5]. Parenchymal lung involvement of the disease is exceedingly rare[2,6].

We report herein a case of unicentric CD, with isolated intrapulmonary involvement and intrafissural development, in a patient who underwent curative surgery.

## CASE REPORT

A 23-year-old, nonsmoking woman, with a familial history of atopy, presented to our department due to a 4-year history of recurring dyspnea and intermittent cough. Chest X-ray film revealed a 4-cm parahilar opacity (Fig. 1). Chest computed tomography (CT) confirmed the presence of a homogenous tissue-density mass, 4.4 × 2.8 cm, located in the right interbronchial space, compressing contiguous vascular and bronchial structures. This mass is associated to subcarinal lymphadenopathies and to infracentimetric lymph nodes located in both right and left laterotracheal chains and in the anterior mediastinum. The mass demonstrated poor contrast enhancement (CE); its origin could not be ruled out, but was most consistent with a voluminous interbronchial lymphadenopathy (Figs. 2A,B).

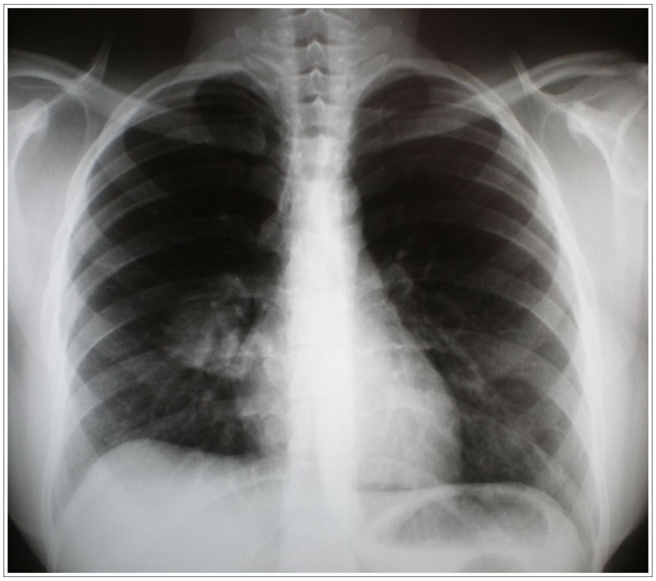

**FIGURE 1.** Chest X-ray film showing a 4-cm parahilar opacity.

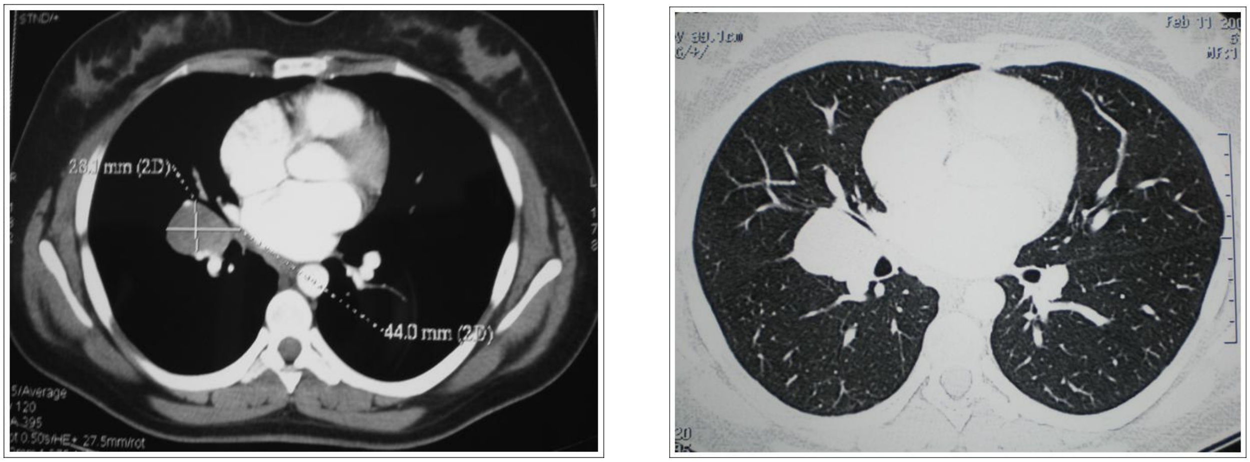

A

B

**FIGURE 2.** CT scan of the chest. (A) Presence of a homogenous tissue-density mass, 4.4 × 2.8 cm, located in the right interbronchial space and presenting poor CE. (B) The mass is compressing contiguous bronchial structures.

Physical examination revealed no abnormalities. Laboratory investigations were within the normal range, except an elevated C-reactive protein rate at 22.6 mg/l. Bronchoscopy showed a bronchial compression at the entry of the right middle lobe.

A right posterior thoracotomy was performed and revealed a 4.5-cm well-defined right hilar mass, with an intrafissural development between the superior and middle lobes. The tumor lesion tightly compressed the middle lobe bronchus and the intrafissural portion of the pulmonary artery. Rapid cytological analysis was intended, but due to profuse bleeding at attempt, and given the tumoral extension to both lobes, a right pneumonectomy was rapidly decided, with total ganglionic subcarinal curage and partial ganglionic resection of the Barety space.

Histopathological examination showed presence of characteristic HV follicles, regularly distributed on a background of lymphocytes (Fig. 3A). The lymphoid follicles present onion-like arranged lymphocytes and interfollicular capillary proliferations. The concentric rings of lymphocytes surround a small atrophic germinal center where an endothelial vascular hyperplasia is observed (Fig. 3B).

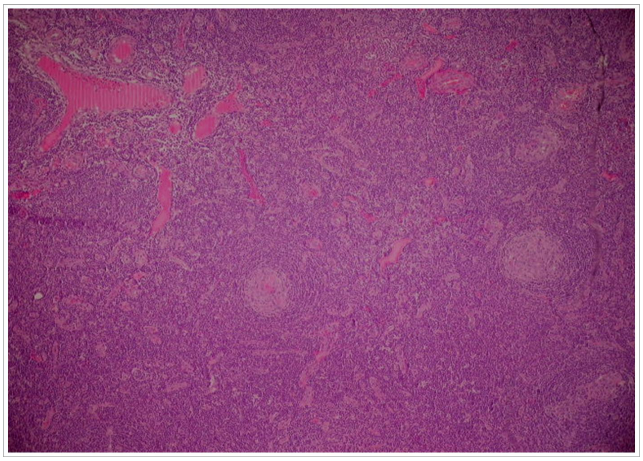

**A**

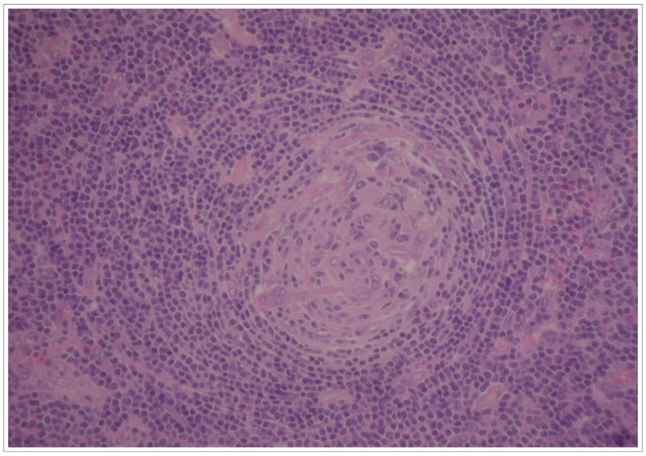

**B**

**FIGURE 3.** Histopathologic examination showing angiofollicular hyperplasia. (A) The characteristic histological pattern is produced by a great number of lymphoid follicles regularly distributed on a background of lymphocytes. (B) A detail of the enlarged lymphoid follicle shows that the central vessels present thick walls and that the endothelial cells are prominent.

A histological diagnosis of benign giant lymph node hyperplasia of the lung corresponding to the HV type of CD was made. Histology of resected lymph nodes was inflammatory. Both HIV-serology and a protein electrophoresis were performed and were normal. The patient has remained well, with no recurrence of symptoms or signs for 1 year.

## DISCUSSION

In this case, unicentric CD presented as a well-defined pulmonary mass, developing in the right interlobar fissure. To our knowledge, isolated parenchymal pulmonary involvement is very rare in CD; a handful of cases have previously been reported. Usually, the disease manifests as a solitary pulmonary nodule[2,6,7,8,9,10,11]. Intrafissural development, as observed in our patient, is an original observation; review of published literature revealed a unique similar case report[12].

The etiology of CD is poorly understood. Danon et al. suggested that the HV type may originate from an antigen stimulus involving a lymph node containing abnormal plasmacytoid monocytes[13].

Unicentric CD has no race or sex predilection and may occur at any age, with a median age at diagnosis of 20 years (range: 8–66 years)[5,14,15]. Clinical and biological manifestations are usually absent; the disease is typically diagnosed through incidental radiological findings in 95% of cases[5,15,16]. The most frequent histological variant encountered in unicentric CD is the HV type[3,5]. The latter is usually asymptomatic, although cases of immunoglobulin and serologic abnormalities have been noted, as well as rare instances of hypochromatic anemia, cytopenia, and thrombocytopenia[17].

CD characteristically affects nodes and originates from the thorax, although the disease can arise wherever lymphoid tissue is found[2]. In up to 15% of cases, extrathoracic involvement is observed and mainly affects the neck, retroperitoneum, and mesentery[5,18]. In 5% of cases, the disease is extranodal[13] and may affect visceral organs, including the liver, spleen, pancreas, and adrenals[19,20], or tissues such as the larynx, parotid gland, various muscles, and cranium[20].

Despite evidence suggesting that thoracic CD usually originates from the lymph nodes along the tracheobronchial tree, some authors found that atypical thoracic localizations (defined as tumors located other than in the mediastinum or pulmonary hilum) accounted for up to 30% of unicentric thoracic CD. Interestingly, only one case of these originated from the lung, which further emphasizes the rarity of isolated intrapulmonary CD[7]. Atypical thoracic localizations of CD include the pleura, axilla, supraclavicular fossa, intercostal space, and pericardium[7,21].

Thoracic unicentric CD typically manifests as a well-defined, rounded, mediastinal or hilar mass of soft tissue density, featuring intense and homogenous contrast enhancement (CE) after medium contrast injection, by virtue of its hypervascular nature[7,14]. However, thoracic CD may arise from unusual localizations[22,23] and occasionally exhibits atypical appearances on CT and MR imaging, as in our patient. Atypical enhancement is defined as poor contrast, target, or concentric enhancement patterns on CT and/or MR imaging[7]. In our case, the mass showed poor CE, which explains why the diagnosis was not considered preoperatively.

Pathologic diagnosis of CD may pose some difficulty. It may mimic other lymphoproliferative disorders in their clinical presentation and morphologic appearance, such as histiocytic sarcoma[24]. In some cases, the latter may be associated with CD, usually the HV type[25].

The surgical approach is usually curative in unicentric CD, but is usually associated with profuse bleeding[26] due to the hypervascularity of these tumors. This propensity to bleed accounts for the difficulty in intraoperative management of CD, and resulted in a total right pneumonectomy in our patient. A few previous reports have reported that preoperative arterial embolization minimized intraoperative bleeding[26] and made the surgical management safer.

CD, despite its rarity and in view of the increasing numbers of reported parenchymal lung involvement of the disease, should be considered in the differential diagnosis of asymptomatic or oligosymptomatic pulmonary nodules.

## ACKNOWLEDGMENT

I would like to acknowledge and thank TheScientificWorldJOURNAL Editor.
